# Emotional distress impairs immune checkpoint blockade efficacy in recurrent high-grade glioma: Insights from tumor in situ fluid analysis

**DOI:** 10.1093/noajnl/vdag040

**Published:** 2026-02-16

**Authors:** Dayang Wang, Jiubing Zhang, Guanzheng Liu, Chaojie Bu, Shaobin Wei, Minghao Li, Guangming Lv, Zhiyuan Sheng, Jie Mei, Zhaoyue Yan, Yushuai Gao, Ruijiao Zhao, Yujie Shi, Meiyun Wang, Xingyao Bu

**Affiliations:** Department of Neurosurgery, Henan University People’s Hospital, Henan Provincial People’s Hospital, Zhengzhou, China; Department of Neurosurgery, Zhengzhou University People’s Hospital, Henan Provincial People’s Hospital, Zhengzhou, China; Clinical Center for Glioma Henan Provincial People’s Hospital, Henan Province Precision Diagnosis and Treatment Engineering Research Center for Glioma, Zhengzhou, China; Department of Neurosurgery, Zhengzhou University People’s Hospital, Henan Provincial People’s Hospital, Zhengzhou, China; Clinical Center for Glioma Henan Provincial People’s Hospital, Henan Province Precision Diagnosis and Treatment Engineering Research Center for Glioma, Zhengzhou, China; Department of Neurosurgery, Zhengzhou University People’s Hospital, Henan Provincial People’s Hospital, Zhengzhou, China; Clinical Center for Glioma Henan Provincial People’s Hospital, Henan Province Precision Diagnosis and Treatment Engineering Research Center for Glioma, Zhengzhou, China; Clinical Center for Glioma Henan Provincial People’s Hospital, Henan Province Precision Diagnosis and Treatment Engineering Research Center for Glioma, Zhengzhou, China; Department of Psychological Medicine, Zhengzhou University People’s Hospital, Henan Provincial People’s Hospital, Henan University People’s Hospital, Zhengzhou, Henan, China; Department of Neurosurgery, Henan University People’s Hospital, Henan Provincial People’s Hospital, Zhengzhou, China; Clinical Center for Glioma Henan Provincial People’s Hospital, Henan Province Precision Diagnosis and Treatment Engineering Research Center for Glioma, Zhengzhou, China; Department of Neurosurgery, Zhengzhou University People’s Hospital, Henan Provincial People’s Hospital, Zhengzhou, China; Clinical Center for Glioma Henan Provincial People’s Hospital, Henan Province Precision Diagnosis and Treatment Engineering Research Center for Glioma, Zhengzhou, China; Department of Neurosurgery, Zhengzhou University People’s Hospital, Henan Provincial People’s Hospital, Zhengzhou, China; Clinical Center for Glioma Henan Provincial People’s Hospital, Henan Province Precision Diagnosis and Treatment Engineering Research Center for Glioma, Zhengzhou, China; Clinical Center for Glioma Henan Provincial People’s Hospital, Henan Province Precision Diagnosis and Treatment Engineering Research Center for Glioma, Zhengzhou, China; Department of Neurosurgery, Zhengzhou University People’s Hospital, Henan Provincial People’s Hospital, Zhengzhou, China; Clinical Center for Glioma Henan Provincial People’s Hospital, Henan Province Precision Diagnosis and Treatment Engineering Research Center for Glioma, Zhengzhou, China; Department of Neurosurgery, Zhengzhou University People’s Hospital, Henan Provincial People’s Hospital, Zhengzhou, China; Clinical Center for Glioma Henan Provincial People’s Hospital, Henan Province Precision Diagnosis and Treatment Engineering Research Center for Glioma, Zhengzhou, China; Department of Neurosurgery, Zhengzhou University People’s Hospital, Henan Provincial People’s Hospital, Zhengzhou, China; Clinical Center for Glioma Henan Provincial People’s Hospital, Henan Province Precision Diagnosis and Treatment Engineering Research Center for Glioma, Zhengzhou, China; Department of Pathology, Henan Provincial People’s Hospital, Zhengzhou, China; Department of Pathology, Henan Provincial People’s Hospital, Zhengzhou, China; Department of Radiology, Henan Provincial People’s Hospital, Zhengzhou, China; Department of Neurosurgery, Henan University People’s Hospital, Henan Provincial People’s Hospital, Zhengzhou, China; Department of Neurosurgery, Zhengzhou University People’s Hospital, Henan Provincial People’s Hospital, Zhengzhou, China; Clinical Center for Glioma Henan Provincial People’s Hospital, Henan Province Precision Diagnosis and Treatment Engineering Research Center for Glioma, Zhengzhou, China

**Keywords:** circulating tumor DNA, emotional distress, high-grade glioma, immune checkpoint blockade, tumor in situ fluid

## Abstract

**Background:**

Immune checkpoint blockade (ICB) therapy has shown limited benefit in recurrent high-grade glioma (HGG), in part due to an immunosuppressive tumor microenvironment. Emotional distress (ED) is known to alter immune regulation, yet its role in shaping the response to ICB in glioma remains unexplored. We aimed to determine the association between pre-treatment ED and ICB efficacy in recurrent HGG (rHGG) and to explore the underlying mechanism using tumor in situ fluid circulating tumor DNA (TISF-ctDNA).

**Methods:**

We prospectively enrolled 75 rHGG patients receiving tislelizumab, bevacizumab, and temozolomide. ED was evaluated using PHQ-9 and GAD-7 before undergoing ICB treatment. Clinical outcomes were compared between ED and No ED groups. TISF-ctDNA sequencing and immunohistochemical staining of tumor tissues were performed to identify pathway alterations and immune markers.

**Results:**

Compared to No ED patients, ED patients had significantly shorter overall survival (15.8 vs. 32.3 months; HR = 2.40, *P* = .006) and progression-free survival (3.4 vs. 7.8 months; *P* = .049), along with lower objective response (10.7% vs. 48.5%, *P* = .002) and clinical benefit rates (39.3% vs. 69.7%, *P* = .017). TISF-ctDNA analysis revealed enrichment of AXON guidance, cAMP signaling, and HPV-related pathways in ED patients. ED was also associated with elevated systemic inflammatory markers (NLR, MLR, PLR; *P* < .05). IHC showed decreased infiltrating immune cells in tumors from ED patients.

**Conclusions:**

Pretreatment ED is associated with impaired ICB efficacy in rHGG, potentially mediated by altered tumor signaling and reduced intratumoral immune cell infiltration. Psychological screening may enhance personalized immunotherapy strategies.

Key Points

- Emotional distress reduces survival and response to ICB in recurrent glioma
- ED correlates with immune suppression and higher systemic inflammation markers
- TISF-ctDNA reveals ED-specific tumor pathways compromising immunotherapy

Importance of the Study

Immune checkpoint blockade (ICB) has limited efficacy in gliomas due to their immunosuppressive microenvironment. While tumor-intrinsic and molecular barriers are well recognized, the role of emotional distress (ED) as an extrinsic, modifiable factor remains unexplored. This study provides the first prospective evidence that pretreatment ED impairs ICB efficacy in recurrent HGG. ED was associated with reduced overall and progression-free survival, lower response rates, elevated systemic inflammation, diminished tumor-infiltrating immune cells, and ED-specific genomic alterations identified via tumor in situ fluid circulating tumor DNA (TISF-ctDNA) profiling. Importantly, No ED patients showed greater ctDNA clearance and immune activation post-treatment. These findings reveal a novel brain-tumor-immune interaction axis through which ED modulates tumor immunity. The results support integrating routine ED screening and psychosocial intervention into glioma immunotherapy protocols, and highlight the translational potential of TISF-guided immune monitoring to personalize treatment strategies.

Immune checkpoint blockade (ICB) therapies have demonstrated remarkable efficacy across numerous cancer subtypes.^1–4^ However, gliomas are classified as “cold” tumors due to their immunosuppressive microenvironment, characterized by few tumor-infiltrating lymphocytes (TILs).^5^^,^^6^ The response to immunotherapy in glioma patients is generally limited, with a minority experiencing prolonged benefits.^7^^,^^8^ A more comprehensive view of optimizing ICB therapy suggests investigating the impact of physiological, environmental, behavioral, and psychological factors (such as diet, physical activity, and mood/mindfulness) on cancer immunotherapy outcomes.^9^ Emotional distress (ED), encompassing negative emotional states in response to stressors, is increasingly recognized as a significant factor.^10^ Cancer patients are at a substantially higher risk of experiencing ED compared to the general population, primarily due to disease-related fears.^11^^,^^12^ Emerging evidence underscores the intricate relationship between emotional and psychological states and host immune function, presenting substantial potential for enhancing cancer treatment, particularly immunotherapy.^13^^,^^14^

The impact of ED on the efficacy of glioma immunotherapy and the progression of the tumor are little known at present. Advances in understanding body-brain neural circuitry reveal its critical role in regulating peripheral inflammation via vagal and brainstem pathways, highlighting the influence of the central nervous system on immune balance.^15^ This framework supports exploring how ED may impact tumor immunity, especially in immunosuppressive “cold” tumors like gliomas. Preclinical studies suggest that ED may facilitate tumor progression and invasiveness by suppressing protective immunity, triggering or exacerbating chronic inflammation, and enhancing immunosuppressive mechanisms.^16^ Additionally, ED triggers the sympatho-adrenal-medullary (SAM) system and the hypothalamic-pituitary-adrenal (HPA) axis, facilitating systemic adaptation. Simultaneously, it influences the peripheral nervous system, encompassing both the sympathetic and parasympathetic divisions, to orchestrate localized reactions. These changes alter the secretion of stress-associated immune-modulating molecules (SAIMs), which interact with immune cell receptors, directly reshaping the tumor’s immune landscape.^17–19^ A study conducted on animals revealed that prolonged stress hastens the advancement of glioblastoma multiforme by activating the DRD2/ERK/β-catenin axis and the positive feedback loop involving Dopamine/ERK/TH.^20^

High-grade glioma (HGG) represents the most common form of primary malignant brain tumor, comprising more than fifty percent of all such tumors.^21^ Individuals with glioma are particularly vulnerable to neurological symptoms, social isolation, and difficulties in interpersonal relationships, more so than patients with other types of cancer.^22^ Moreover, the fear associated with HGG exacerbates patients’ vulnerability to ED.^23^ Currently, because ICB therapy is not a standard glioma treatment, no studies have explored the association between ED and ICB response in this population, underscoring the urgent need for prospective clinical investigations.

This study presents the findings of a prospective observational study exploring the association between ED and ICB response in recurrent HGG (rHGG), and investigates potential mechanisms via tumor in situ fluid (TISF), which reflects the tumor’s in vivo environment.^24–27^

## Methods

### Study Design Study Population

This prospective, single-center, observational clinical study aims to investigate the correlation between ED and clinical outcomes of ICB therapy in rHGG patients. Initiated on June 1, 2021, the study enrolled 75 glioma patients between June 1, 2021, and May 16, 2024 (NCT05515133). Please see Supplementary Methods for the inclusion and exclusion criteria. The primary objective was to evaluate the relationship between ED and ICB therapy efficacy in rHGG. The primary endpoint was OS, with secondary endpoints including objective response rate (ORR), clinical benefit rate (CBR), and PFS. Exploratory outcomes included quality of life (QoL) assessments, DNA sequencing analysis, and TILs by immunohistologic analysis (see Supplementary Methods). Ethical approval for this investigation was obtained from the Ethics Committee of Henan Provincial People’s Hospital. Informed consent was acquired in writing from all participants, and treatments were administered at the same institution. Baseline data were recorded before the initiation of immunotherapy. Each patient received intravenous tislelizumab (BeiGene, Ltd.) 200 mg and bevacizumab (Roche Pharma Ltd.) 3 mg/kg every 3 weeks, in combination with their standard temozolomide (Beijing Sl Pharmaceutical Co., Ltd.) regimen, until either radiological signs of progression or intolerable side effects were observed.

### Follow-Up and Progression Status

Patients underwent baseline brain MRI with contrast enhancement within 1 week prior to their first ICB therapy session as a reference for comparison. Imaging follow-ups occurred every 3-5 weeks until secondary tumor progression was detected. Tumor response was evaluated by two authors, Dr. Wang and Dr. Zhang, following the Response Assessment in Neuro-Oncology 2.0 (RANO 2.0) criteria.^28^ For patients exhibiting disease progression, the immunotherapy-specific RANO (iRANO) criteria were applied. OS was defined as the duration from the initial surgery until death from any cause. PFS was the time from the first ICB treatment to either disease progression or death, whichever occurred sooner. Although enrollment was completed, patient follow-up remained ongoing. Data collection concluded on June 30, 2024.

### Evaluation of Emotional Distress

In line with the American Society of Clinical Oncology’s guidelines, the evaluation of anxiety and depression was performed using the Patient Health Questionnaire (PHQ-9) for depression and the Generalized Anxiety Disorder (GAD-7) scale to assess anxiety.^29^ The PHQ-9 includes nine questions designed to evaluate depressive symptoms experienced over the past 2 weeks, with total scores ranging from 0 to 27.^30^ The GAD-7, consisting of seven items, measures anxiety symptoms within the same 2-week period, with scores ranging from 0 to 21.^31^ In this study, ED was defined as a score of 5 or higher on either the PHQ-9 or GAD-7. The severity of ED was classified as follows: (1) No ED: Scores on both the PHQ-9 and GAD-7 fall between 0 and 4; (2) Mild ED: Scores of 5 to 9 on either the PHQ-9 or GAD-7; (3) Severe ED: Scores of 10 or higher on either the PHQ-9 or GAD-7. Patients were evaluated within 2 weeks prior to commencing immunotherapy.

### TISF and Peripheral Blood Collection

TISF and peripheral blood samples were obtained and processed at Henan Provincial People’s Hospital. To enable postoperative collection of TISF, a fluid reservoir capsule was implanted intraoperatively at the time of the initial diagnostic surgery by experienced neurosurgeons using neuronavigation guidance, and was placed between the periosteum and the galea aponeurosis. Placement was performed under a strict aseptic protocol with prophylactic antibiotic administration according to institutional practice. Because this study enrolled patients at first recurrence, the reservoir had already been in place prior to study entry, and no additional surgical procedure was performed specifically for reservoir implantation. Samples were collected at each treatment cycle after evidence of disease progression, with baseline samples taken prior to the start of treatment. Patients provided 0.5 to 1 ml of TISF via the implanted reservoir. Additionally, 5 ml of peripheral blood was drawn to analyze germline DNA and measure circulating biomarkers. Plasma was separated through Ficoll-Hypaque density gradient centrifugation at 1500 rpm for 5 minutes to remove cellular components. The supernatant was stored at −80°C for subsequent analysis. Similarly, TISF samples underwent the same processing steps, with their supernatants stored at −80°C. Systemic inflammation markers, such as the Neutrophil-to-Lymphocyte Ratio (NLR), Monocyte-to-Lymphocyte Ratio (MLR), and Platelet-to-Lymphocyte Ratio (PLR), were assessed through routine blood tests. These markers were categorized based on their median values and analyzed for potential correlations with clinical characteristics and patient survival.

### Statistical Analysis

Statistical analyses were carried out using IBM SPSS software, version 23 (IBM Corp., Armonk, NY, USA). Differences between categorical variables were examined using Pearson’s chi-square test, with Fisher’s exact test employed for small sample sizes. For continuous variables, either independent-samples t-tests or nonparametric alternatives (Mann-Whitney for independent data and Wilcoxon for paired data) were applied, depending on distribution patterns. Survival analysis was conducted with the Kaplan-Meier method, and comparisons were made using the log-rank test and Gehan-Breslow-Wilcoxon test to assess the association between ED and primary or secondary outcomes. The relationship between ED and survival outcomes was evaluated using Cox proportional hazards regression, with multivariable Cox models incorporating clinical prognostic factors. Variables achieving statistical significance (*P* < .05) in univariate analysis were included in the multivariable models. To ensure balanced comparison groups, propensity score matching (PSM) was implemented.^32^ This process matched ED patients to No ED patients based on a multivariable logistic regression model, where ED status was treated as the outcome variable. Matching utilized a greedy nearest neighbor algorithm without replacement, applying a caliper width of 0.02 and a 1:1 ratio. Sensitivity analyses further assessed the relationship between ED severity and clinical outcomes, focusing on OS.

## Results

### Patients and Baseline Characteristics

**Figure 1.**
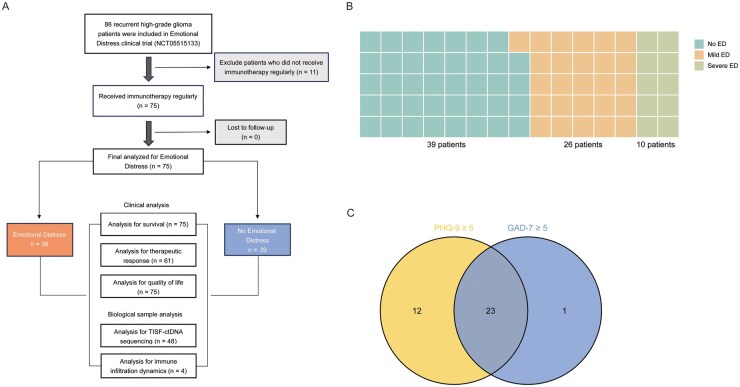
Participant Flowchart and the Distribution of ED. (A) Participant flowchart illustrating patient enrollment, inclusion, and exclusion criteria for the ED clinical trial (NCT05515133). A total of 86 patients with recurrent HGG were screened, 75 patients received regular immunotherapy and were included in the final analysis. Patients were stratified into two groups: ED (n = 36) and No ED (n = 39). Analyses included survival outcomes, therapeutic response, quality of life, TISF-ctDNA sequencing, and immune infiltration dynamics. (B) Stratification of patients based on ED severity. Among patients with emotional distress, 26 exhibited mild ED and 10 exhibited severe ED. (C) Distribution of individual PHQ-9 and GAD-7 item scores in the cohort. Among 36 patients with ED, 12 exhibited exclusively PHQ-9 scores ≥5, 1 had solely GAD-7 scores ≥5, and 23 presented with both PHQ-9 and GAD-7 scores ≥5. Abbreviations: ED: emotional distress; TISF-ctDNA: tumor in situ fluid-circulating tumor DNA.

By May 16, 2024, a total of 178 patients were screened for inclusion in the study. Of these, 92 patients (51.7%) were excluded prior to enrollment due to reasons including a diagnosis of low-grade glioma, irregular adherence to first-line treatment following surgery, loss of communication or literacy skills before the initiation of immunotherapy, and refusal to undergo immunotherapy after relapse. Consequently, 86 patients were enrolled in the study. After enrollment, 11 patients (12.8%) were further excluded for not consistently receiving immunotherapy post-relapse or for undergoing fewer than two sessions of immunotherapy. Ultimately, 75 patients were included in the analysis investigating the impact of ED on immune checkpoint blockade response in rHGG. The study design and patient selection process are depicted in Figure 1A. All patients completed an ED assessment within 2 weeks before initiating immunotherapy. The median follow-up duration was 14.9 months (95% CI: 11.9-19.1). The median age was 55 years, and 58.7% (44/75) of patients were men. According to the 2021 WHO CNS classification, tumors were categorized as glioblastoma, IDH-wildtype (80.0%, 60/75), astrocytoma, IDH-mutant (16.0%, 12/75), or oligodendroglioma, IDH-mutant (4.0%, 3/75). MGMT promoter methylation was present in 37.3% (28/75), absent in 45.3% (34/75), and not detected in 17.3% (13/75). Baseline characteristics are summarized in Table 1. At baseline, 48.0% of patients (36/75) were identified with ED, with 72.2% (26/36) classified as mild ED and 27.8% (10/36) as severe ED (Figure 1B). The distribution of individual PHQ-9 and GAD-7 item scores in the cohort is shown in Figure 1C. Multivariate logistic regression revealed that patients with lower baseline Karnofsky performance score (KPS) (70-80) was more likely to experience ED (Supplementary Table S1).

**Table 1.** Baseline Characteristics of 75 Enrolled Patients

Characteristics		No ED (*n* = 39)	ED (*n* = 36)	Total (*n* = 75)	*P* value
Age (years), *n* (%)	<55	19(48.7)	18(50.0)	37(49.3)	.912
	≥55	20(51.3)	18(50.0)	38(50.7)	
Sex, *n* (%)	Male	20(51.3)	24(66.7)	44(58.7)	.176
	Female	19(48.7)	12(33.3)	31(41.3)	
KPS, *n* (%)	70-80	10(25.6)	17(47.2)	27(36.0)	.052
	90-100	29(74.4)	19(52.8)	48(64.0)	
Histopathologic diagnosis, *n* (%)	Glioblastoma, IDH-wildtype	30(76.9)	30(83.3)	60(80.0)	.361^a^
	Astrocytoma, IDH-mutant	6(15.4)	6(16.7)	12(16.0)	
	Oligodendroglioma, IDH-mutant	3(7.7)	0(0.0)	3(4.0)	
Primary tumor location, *n* (%)	Frontal lobe	17(43.6)	14(38.9)	31(41.3)	.828^a^
	Temporal lobe	10(25.6)	13(36.1)	23(30.7)	
	Parietal lobe	7(17.9)	6(16.7)	13(17.3)	
	Thalamus	3(7.7)	1(2.8)	4(5.3)	
	Occipital lobe	1(2.6)	1(2.8)	2(2.7)	
	Insular lobe	1(2.6)	0(0.0)	1(1.3)	
	Brainstem	0(0.0)	1(2.8)	1(1.3)	
MGMT promoter methylation, *n* (%)	No	17(43.6)	17(47.2)	34(45.3)	.951
	Yes	15(38.5)	13(36.1)	28(37.3)	
	Not-detected	7(17.9)	6(16.7)	13(17.3)	
Radiation, *n* (%)	No	23(59.0)	27(75.0)	50(66.7)	.141
	Yes	16(41.0)	9(25.0)	25(33.3)	
Tumor size at baseline (mm)	Median	27.3	25.9	26.3	.265^b^
	Range	8.5-77.6	8.9-81.9	8.5-81.9	
Baseline steroid use, *n* (%)	No	32(82.1)	27(75.0)	59(78.7)	.456
	Yes	7(17.9)	9(25.0)	16(21.3)	
Extent of surgery, *n* (%)	Complete resection	28(71.8)	22(61.1)	50(66.7)	.390^a^
	Partial resection	11(28.2)	13(36.1)	24(32.0)	
	Unable to assess	0(0.0)	1(2.8)	1(1.3)	
Smoking, *n* (%)	No	31(79.5)	23(63.9)	54(72.0)	.133
	Yes	8(20.5)	13(36.1)	21(28.0)	
Hypertension/diabetes, *n* (%)	No	26(66.7)	24(66.7)	50(66.7)	1.000
	Yes	13(33.3)	12(33.3)	25(33.3)	
Educational level, *n* (%)	<High school	17(43.6)	14(38.9)	31(41.3)	.680
	≥High school	22(56.4)	22(61.1)	44(58.7)	
Marital status, *n* (%)	Married	1(2.6)	5(13.9)	6(8.0)	.099^a^
	Others	38(97.4)	31(86.1)	69(92.0)	
Job status, *n* (%)	Unemployed	31(79.5)	30(83.3)	61(81.3)	.669
	Employed	8(20.5)	6(16.7)	14(18.7)	
Caregiver, *n* (%)	Spouse	17(43.6)	15(41.7)	32(42.7)	.866
	Others	22(56.4)	21(58.3)	43(57.3)	
Residence, *n* (%)	Country	27(69.2)	28(77.8)	55(73.3)	.403
	City	12(30.8)	8(22.2)	20(26.7)	
Monthly income (CNY), *n* (%)	<5,000	30(76.9)	26(72.2)	56(74.7)	.640
	≥5,000	9(23.1)	10(27.8)	19(25.3)	
BMI (kg/m^2^), *n* (%)	<24	22(56.4)	18(50.0)	40(53.3)	.578
	≥24	17(43.6)	18(50.0)	35(46.7)	

a The histopathologic diagnosis, primary tumor location, extent of surgery, and marital status were analyzed using two-sided Fisher’s exact tests, and the proportions of all other categorical characteristics were analyzed using two-sided χ^2^ tests.

b Tumor size at baseline was compared between groups using the Mann-Whitney U test.

### ED Was Associated with a Reduction in OS

**Figure 2.**
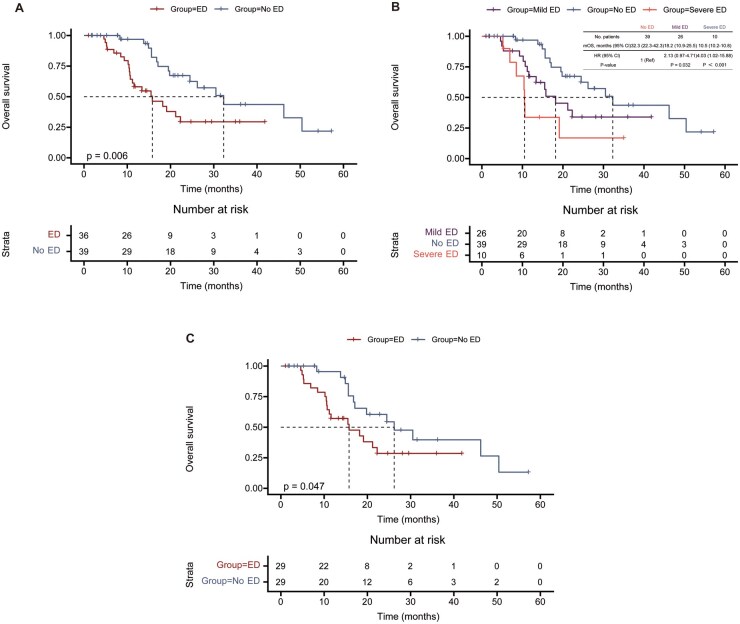
Kaplan-Meier Survival Analysis of OS Stratified by ED and Its Severity. (A) Kaplan-Meier curve of OS comparing ED patients and No ED patients in the entire cohort. ED patients showed markedly reduced survival. (B) Kaplan-Meier curve of OS stratified by ED severity (No ED, mild ED, and severe ED). Both mild and severe ED patients showed significantly reduced OS compared to No ED patients. (C) Kaplan-Meier curve of OS in the PSM cohort comparing No ED and ED groups. The survival disadvantage in ED patients remained significant after adjustment.

By June 30, 2024, a total of 36 OS events were documented. The median OS for the entire cohort was 21.2 months (95% CI: 14.1-28.3). Patients in the No ED group demonstrated a median OS of 32.3 months (95% CI: 22.3-42.3), while the ED group had a median OS of 15.8 months (95% CI: 8.4-23.1). Survival analysis revealed that OS was significantly shorter in the ED group compared to the No ED group under immunotherapy (hazard ratio [HR] 2.40, 95% CI: 1.22-4.72; *P* = .006, Figure 2A). To further investigate the association between ED and OS, both univariate and multivariate Cox regression analyses were performed. Univariate analysis identified significant associations between OS and variables including age, KPS, *IDH-1* mutation status, baseline steroid use, caregiver, and ED status. However, given the potential for rare event bias and the complete separation observed for *IDH-1* mutation status, Firth’s penalized multivariate Cox regression was employed to provide unbiased parameter estimates. Based on these findings, age, KPS, *IDH-1* mutation status, baseline steroid use, caregiver, and ED status were included in the Firth-adjusted multivariate Cox regression analysis. This analysis identified ED ([HR] 2.74, 95% CI: 1.27-5.92; *P* = .01) and age ≥55 years ([HR] 4.74, 95% CI: 1.87-12.05; *P* = .001) as independent predictors of poorer OS, while *IDH-1* mutation was a favorable prognostic factor ([HR] 0.13, 95% CI: 0.02-0.78; *P* = .026, Supplementary Figure S1). To create balanced cohorts, propensity score matching (PSM) was employed, matching ED patients to No ED patients. This process resulted in 59 matched patients, with 28 in each group. In the PSM sample, baseline characteristics were well balanced (Supplementary Table S2). Thirty-one OS events were observed, with the No ED group having a median OS of 26.2 months (95% CI: 14.3-38.1) versus 15.8 months (95% CI: 6.8-24.8) in the ED group. OS was significantly lower in the ED group (HR 1.98, 95% CI: 0.97-4.05; *P* = .047, Figure 2C). We then explored the association between the severity of ED and OS. The median OS for patients with mild ED was 18.2 months (95% CI 10.9 to 25.5), while for those with severe ED it was 10.5 months (95% CI 10.2 to 10.8). Compared to the No ED group, both mild (HR 2.13, 95% CI: 0.97-4.71; *P* = .03) and severe ED (HR 4.03, 95% CI: 1.02-15.88; *P* < .001) were associated with significantly shorter OS (Figure 2B).

### ED Is Associated with Lower Objective Response Rate and Clinical Benefit Rate

**Figure 3.**
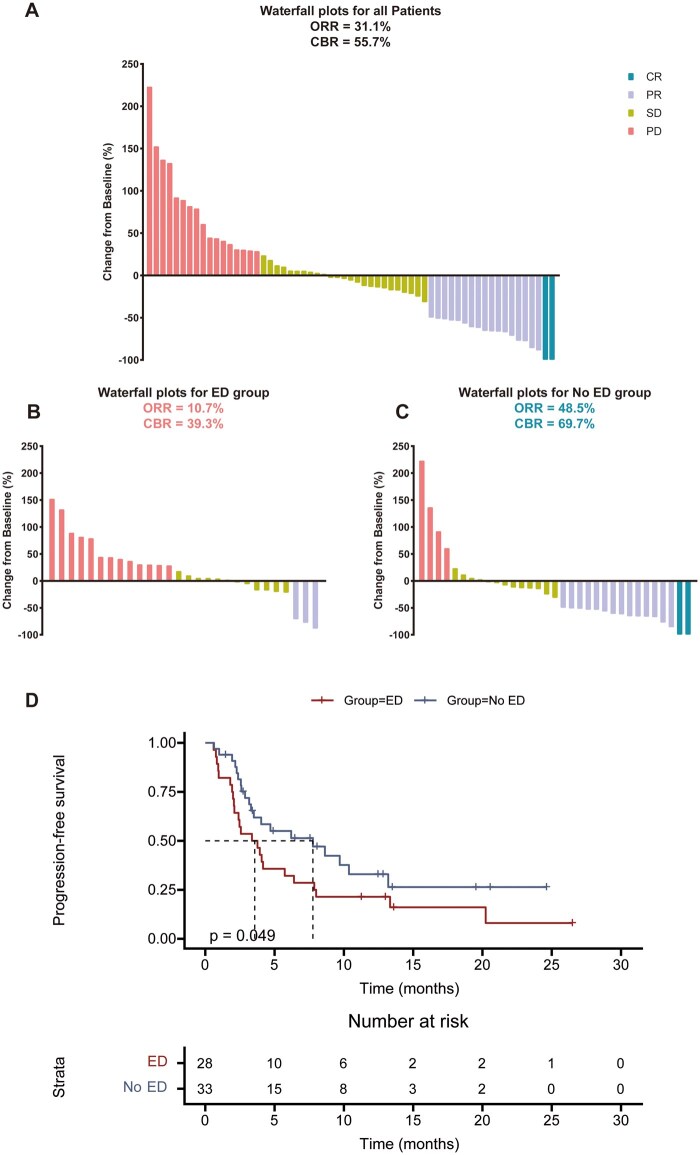
Therapeutic Response and PFS Stratified by ED. (A-C) Waterfall plots showing maximum percentage change in target lesion size from baseline in (A) all patients, (B) No ED patients, and (C) ED patients. (D) Kaplan-Meier curve for PFS stratified by ED status. Patients with ED had significantly shorter PFS compared to those without ED. The *P*-value was determined by Gehan-Breslow-Wilcoxon test. PFS: progression-free survival; CR: complete response; PR: partial response; SD: stable disease; PD: progressive disease; ORR: overall response rate; CBR: clinical benefit rate.

We analyzed the relationship between ED and secondary endpoints, including the objective response rate (ORR), clinical benefit rate (CBR), and PFS in the context of immunotherapy. As of the data cutoff, imaging evaluations revealed 2 patients with a complete response (CR), 17 with a partial response (PR), 25 exhibiting stable disease (SD), and 17 showing progressive disease (PD). 14 patients had no evaluable post-ICB imaging data. The overall ORR (CR + PR) was 31.1%, with 48.5% in the No ED group vs. 10.7% in the ED group. The overall CBR (CR/PR/SD ≥6 months) was 55.7%, with 69.7% in the No ED group compared to 39.3% in the ED group. Figure 3A-C illustrate the maximum percentage change in target lesion size from baseline for both groups following immunotherapy. The ED group demonstrated a significantly lower probability of achieving ORR (48.5% vs. 10.7%, odds ratio [OR] 0.13; 95% CI: 0.03-0.51; *P* = .002) and CBR (69.7% vs. 39.3%, OR 0.28; 95% CI: 0.10-0.81; *P* = .017) compared to the No ED group. By the data cutoff, 44 progression-free survival (PFS) events had been documented. The median PFS for the entire cohort was 4.1 months (95% CI: 1.8-6.4). Patients in the No ED group demonstrated a median PFS of 7.8 months (95% CI: 2.1-13.4), while the ED group had a median PFS of 3.4 months (95% CI: 1.5-5.3). PFS was notably shorter in the ED group compared with the No ED group (*P* = .049, Figure 3D).

### Patients with ED Performed Worse on Several Aspects of Assessment of QoL

Supplementary Table S3 presented baseline EORTC-QLQ-C30 scores for the No ED and ED groups. Compared to the No ED group, the ED group had significantly lower scores in Global health (median [IQR]: 83.3 [66.7-83.3] vs. 50.0 [35.4-58.3]; *P* < .001), Physical functioning (86.7 [80.0-93.3] vs. 63.3 [46.7-80.0]; *P* < .001), Role functioning (100.0 [66.7-100.0] vs. 66.7 [50.0-95.8]; *P* < .001), Emotional functioning (91.7 [83.3-100.0] vs. 75.0 [58.3-83.3]; *P* < .001), Cognitive functioning (66.7 [66.7-83.3] vs. 50.0 [33.3-66.7]; *P* < .001), Social functioning (83.3 [66.7-100.0] vs. 50.0 [33.3-66.7]; *P* < .001), Fatigue (33.3 [22.2-33.3] vs. 33.3 [33.3-66.7]; *P* = .001), Nausea and vomiting (0.0 [0.0-0.0] vs. 0.0 [0.0-29.2]; *P* = .043), Pain (0.0 [0.0-16.7] vs. 16.7 [0.0-33.3]; *P* = .010), Insomnia (0.0 [0.0-33.3] vs. 33.3 [0.0-33.3]; *P* = .008), Appetite loss (0.0 [0.0-33.3] vs. 33.3 [0.0-33.3]; *P* = .002), and Financial difficulties (33.3 [33.3-66.7] vs. 66.7 [33.3-100.0]; *P* < .001). Scores for dyspnea (0.0 [0.0-0.0] vs. 0.0 [0.0-33.3]; *P* = .141), Constipation (0.0 [0.0-33.3] vs. 0.0 [0.0-33.3]; *P* = .580), and Diarrhea (0.0 [0.0-0.0] vs. 0.0 [0.0-0.0]; *P* = .881) were similar between the groups.

### ED Alters Key Molecular Pathways and Genetic Landscapes, Potentially Compromising Immunotherapy Efficacy in High-Grade Gliomas

**Figure 4.**
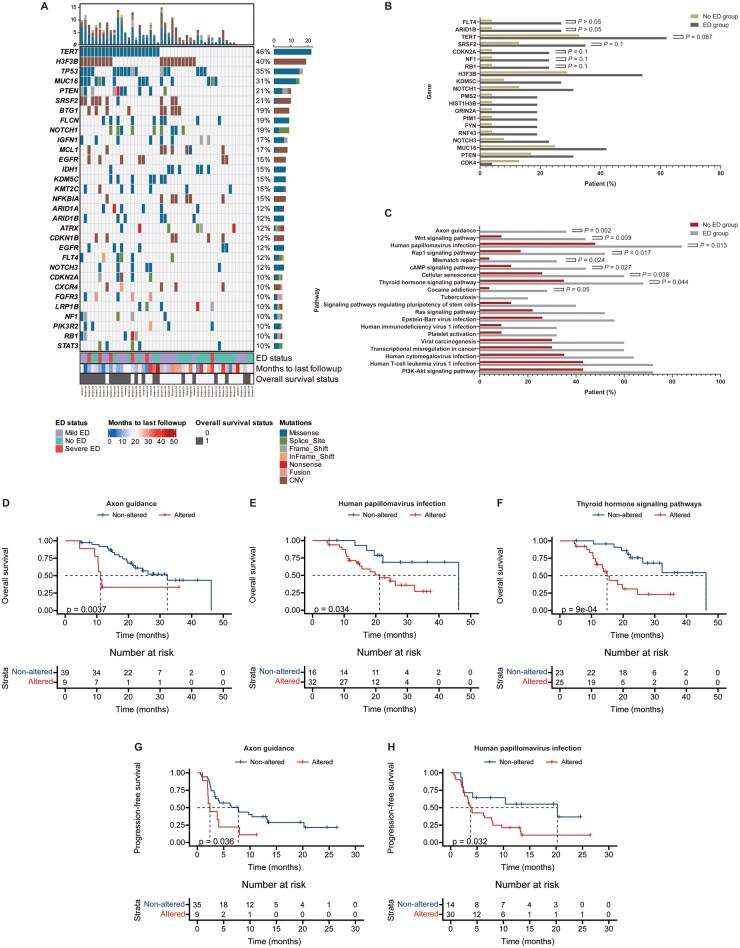
Genomic and Pathway Alterations with Survival Analysis Stratified by ED. (A) Oncoplot of somatic alterations in baseline TISF-ctDNA from 48 patients with recurrent high-grade glioma. Bar plots summarize mutation types and frequency per gene. (B) Comparison of mutation frequencies for specific genes between ED and No ED groups. (C) Pathway enrichment analysis showing altered pathways between ED and No ED groups. (D-F) Kaplan-Meier overall survival curves for patients with and without alterations in (D) Axon guidance, (E) human papillomavirus infection, and (F) thyroid hormone signaling pathways. All three alterations were associated with significantly shorter overall survival. (G-H) Kaplan-Meier analysis of PFS stratified by somatic alterations in key signaling pathways. (G) Patients with alterations in the Axon guidance pathway showed significantly shorter PFS compared to those without alterations. (H) Patients with alterations the Human papillomavirus infection pathway were associated with reduced PFS. The *P*-value was determined by Log-rank test. See also Supplementary Figure S2.

Genetic landscape differences and pathway alterations may elucidate mechanisms underlying the reduced immunotherapy efficacy in ED patients. Given the temporal heterogeneity of gliomas, initial tumor tissue may not accurately reflect recurrent disease characteristics. To address this, we developed a novel liquid biopsy method and defined a new liquid, TISF, collected directly from the TISF collector. Our previous studies demonstrated that TISF can provide real-time insights into tumor genetic evolution and characteristics, which contains a higher concentration of circulating tumor DNA (ctDNA) than that found in blood or cerebrospinal fluid. Among 48 patients with baseline TISF samples, 93.8% exhibited at least one genomic alteration at baseline. In both subgroups, *TERT* and *TP53* were identified as the most commonly mutated genes. Besides, amplifications of *H3F3B* and *MUC16* were observed at high frequency in patients with or without ED (Figure 4A). Mutation frequency analysis at the gene level revealed no significant differences between groups (Figure 4B). Gene set enrichment analysis utilizing the Kyoto Encyclopedia of Genes and Genomes (KEGG) pathways database revealed that somatic genetic alterations associated with AXON guidance, wnt signaling pathway, human papillomavirus infection, rap1 signaling pathway, mismatch repair, cAMP signaling pathway, cellular senescence, thyroid hormone signaling pathway and cocaine addiction were observed more frequently in ED patients, while other carcinogenic pathways showed no significant differences (Figure 4C). Subsequent survival analysis of 48 patients with baseline TISF samples revealed that somatic alterations in AXON guidance (HR 3.53, 95% CI: 0.85-14.61; *P* = .004), human papillomavirus infection (HR 2.69, 95% CI: 1.18-6.15; *P* = .034), and thyroid hormone signaling pathways (HR 3.69, 95% CI: 1.55-8.75; *P* < .001) were significantly associated with shorter OS (Figure 4D-F). Analysis of 44 patients with baseline TISF samples and evaluable radiologic data showed that somatic alterations in AXON guidance (HR 2.27, 95% CI: 0.80-6.40; *P* = .036) and human papillomavirus infection pathways (HR 2.38, 95% CI: 1.17-4.83; *P* = .032) correlated with reduced PFS compared to patients without these alterations (Figure 4G-H). Additionally, patients with alterations in AXON guidance (45.7% vs. 0%; *P* = .016) and cAMP signaling pathways (46.7% vs. 14.3%; *P* = .038) exhibited lower ORR. No substantial differences were found in the additional pathways that were evaluated (Supplementary Figure S2A and B). These findings suggest that ED may modulate immunotherapy outcomes by altering pathways such as AXON guidance. Further studies are necessary to delineate causal relationships and elucidate the precise molecular mechanisms underlying these associations.

### ED Impairs Immune Activation and Associates with TMB and Systemic Inflammation in Recurrent Glioma

**Figure 5.**
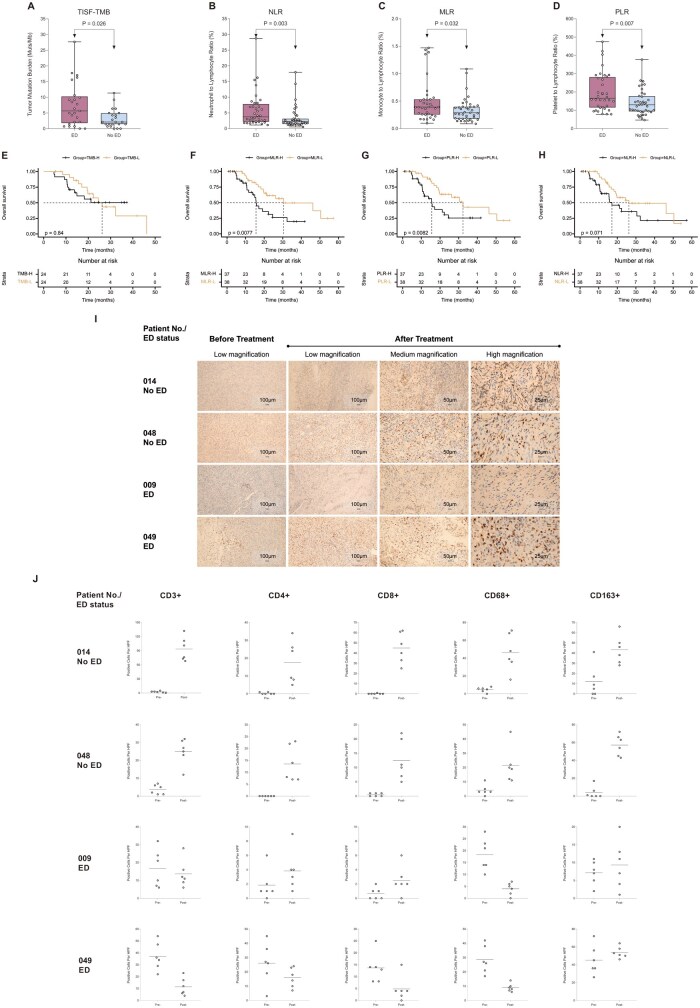
Association of Tumor Mutational Burden and Systemic Inflammatory Markers with Emotional Distress and Survival Outcomes. (A) TISF-TMB at baseline was significantly higher in patients with ED compared to those without ED. (B-D) Systemic inflammatory markers in peripheral blood at baseline, including (B) neutrophil-to-lymphocyte ratio, (C) monocyte-to-lymphocyte ratio, and (D) platelet-to-lymphocyte ratio, were all significantly elevated in ED patients. (E-H) Kaplan-Meier survival analysis of OS stratified by baseline (E) TMB, (F) MLR, (G) PLR, and (H) NLR. High MLR and PLR were significantly associated with shorter OS. Differences in OS between high and low TMB or NLR groups were not statistically significant. (I) Representative immunohistochemical staining of CD163+ macrophages in pre- and post-treatment tumor tissues from four patients. Post-treatment tissues from all four patients exhibited a notable increase in CD163+ macrophage infiltration compared to their pre-treatment counterparts. Images were acquired at low, medium, and high magnifications as indicated. Scale bars: 100 μm, 50 μm, 25 μm. (J) Quantification of immune cell infiltration per high-power field before and after treatment in patients 014, 048 (No ED), and 009, 049 (ED). Positive cells were manually counted in six randomly selected tumor invasion front fields for each marker. Bars represent means. Post-treatment tissues from No ED patients showed increased infiltration of CD3^+^, CD4^+^, CD8^+^, CD68^+^, and CD163^+^ immune cells. In contrast, ED patients exhibited a general decrease in immune cell infiltration, with the exception of CD163^+^ macrophages and slight increases in CD4^+^ and CD8^+^ T cells, though these changes were minimal. The *P*-value was determined by Log-rank test. TMB-H: tumor mutational burden-high; TMB-L: tumor mutational burden-low; NLR-H: neutrophil-to-lymphocyte ratio-high; NLR-L: neutrophil-to-lymphocyte ratio-low; MLR-H: monocyte-to-lymphocyte ratio-high; MLR-L: monocyte-to-lymphocyte ratio-low; PLR-H: platelet-to-lymphocyte ratio-high; PLR-L: platelet-to-lymphocyte ratio-low.

To elucidate mechanisms underlying the association between ED and ICB response, we evaluated baseline TISF-TMB levels as a potential marker of tumor aggressiveness. Among 48 patients with baseline TISF samples, the ED group exhibited significantly higher TISF-TMB levels compared to the No ED group (median [IQR]: 5.7 [1.8-10.3] vs. 2.1 [1.4-5.0] Muts/Mb; *P* = .026, Figure 5A). Using a median TMB threshold of 3.9 Muts/Mb, no statistically significant difference in OS was detected between the low and high TISF-TMB groups (undefined [95% CI: 11.2-undefined] vs. 26.2 [95% CI: 17.1-46.2] months; HR 1.09, 95% CI: 0.48-2.47; *P* = .84, Figure 5E). Next, baseline immune cell subset ratios in peripheral blood were also assessed. Peripheral blood was collected from all patients (75/75) at baseline. Results indicated that patients with ED exhibited significantly higher baseline neutrophil-to-lymphocyte ratio (NLR) (median [IQR]: 3.7 [2.1-7.8] vs. 2.2 [1.4-3.2]; *P* = .003, Figure 5B), monocyte-to-lymphocyte ratio (MLR) (median [IQR]: 0.4 [0.3-0.5] vs. 0.3 [0.2-0.4]; *P* = .03, Figure 5C), and platelet-to-lymphocyte ratio (PLR) (median [IQR]: 165.9 [114.8-281.7] vs. 130.0 [93.5-177.9]; *P* = .007) compared to No ED patients (Figure 5D). Using median values as cutoffs, high baseline MLR (30.5 [95% CI: 19.5-undefined] vs. 15.6 [95% CI: 11.6-22.3] months; HR 2.33, 95% CI: 1.17-4.62; *P* = .008, Figure 5F) and PLR (32.3 [95% CI: 19.5-50.4] vs. 15.6 [95% CI: 10.6-22.3] months; HR 2.32, 95% CI: 1.17-4.59; *P* = .008, Figure 5G) were associated with poorer OS. Although baseline NLR was not significantly associated with OS (26.2 [95% CI: 18.2-50.4] vs. 17.1 [95% CI: 10.7-30.5] months; HR 1.80, 95% CI: 0.92-3.53; *P* = .07, Figure 5H), NLR may have potential prognostic value. In parallel, immunohistochemical analysis of paired pre- and post-treatment tumor tissues from four patients undergoing a second surgical resection due to treatment-resistant progression revealed distinct immune infiltration dynamics stratified by ED status. Patients 014 and 048 were classified as No ED, while patients 009 and 049 were classified as ED. Overall, post-treatment tissues from all four patients exhibited a notable increase in CD163+ macrophage infiltration compared to their pre-treatment counterparts (Figure 5I). Notably, differential immune responses emerged upon subgroup analysis by ED status: patients without ED (014 and 048) demonstrated an increase in infiltrating immune cells (CD3+, CD4+, CD8+, CD68+, and CD163+) post-treatment, indicative of enhanced immune activation. Conversely, among the ED patients, patient 049 showed a universal decrease in all immune cell populations except CD163+ macrophages. Patient 009 exhibited reduced infiltration of CD3+ T cells and CD68+ macrophages post-treatment; although slight median increases were observed for CD4+ and CD8+ T cells, these counts remained low both pre- and post-treatment, with minimal variation **(**Figure 5J**)**. These findings underscore a potential mechanistic association between ED and impaired antitumor immune responses in rHGG patients receiving immunotherapy.

### No ED is Associated with a Reduction in TISF-ctDNA MVAF following ICB Treatment

Next, we analyzed the association between ED status and TISF-ctDNA. Among the 48 patients with baseline TISF, 62.5% (30/48) had serial TISF samples. The analysis identified no notable difference in baseline TISF-ctDNA Maximal Somatic Variant Allelic Frequency (MVAF) between ED and No ED groups (median [IQR]: 5.3 [3.1-17.5] vs. 4.4 [2.8-21.7]; *P* = .98, Supplementary Figure S3A). Furthermore, no significant association with OS was observed (19.8 [95% CI: 11.6-46.2] vs. 24.5 [95% CI: 19.5-undefined] months; HR 0.82, 95% CI: 0.36-1.86; *P* = .63, Supplementary Figure S3B). Further analysis of TISF-ctDNA data from patients with detectable baseline TISF-ctDNA and serial TISF-ctDNA NGS (N = 30) indicated that ED patients were less likely than No ED patients to experience a decrease in TISF-ctDNA MVAF following ICB treatment (40.0% vs. 80.0%; OR 0.17; 95% CI: 0.03-0.85; *P* = .025, Supplementary Figure S3C). Additionally, patients with a decrease MVAF post-ICB treatment (N = 18) had significantly longer OS compared to those with an increase (N = 12) (32.3 [95% CI: 24.5-46.2] vs. 19.5 [95% CI: 9.3-undefined] months; HR 2.89, 95% CI: 0.87-9.60; *P* = .040, Supplementary Figure S3D). We identified 4 patients who cleared TISF-ctDNA post-treatment and 26 patients with detectable TISF-ctDNA. The median OS for patients who cleared TISF-ctDNA after ICB treatment was undefined months (95% CI: 24.5-undefined), compared to 32.3 months (95% CI: 18.2-46.2; *P* = .43) in those with detectable ctDNA (Supplementary Figure S3E). Notably, 100% (4/4) of patients who cleared TISF-ctDNA post-treatment were in the No ED group, suggesting that No ED status may be associated with a higher likelihood of molecular-level disease remission.

## Discussion

This observational study prospectively explored the association between ED and clinical outcomes in patients with rHGG receiving ICB therapy. As far as we know, this represents the largest prospective investigation of the association between ED and ICB response in rHGG. The results indicate that patients experiencing ED prior to ICB treatment exhibited significantly reduced OS and PFS compared to those without ED. Furthermore, these patients demonstrated lower ORR and CBR, underscoring the substantial influence of psychological factors on treatment efficacy. The study also highlights the utility of TISF in monitoring glioma progression, particularly in ED patients. By analyzing TISF-ctDNA, we observed a distinct mutational landscape that may contribute to the observed reduced efficacy of ICB in ED patients. TISF provides a comprehensive and sensitive representation of tumor genetic evolution, allowing for real-time monitoring of residual disease and offering insights into tumor dynamics not captured by traditional tissue biopsies.

Previous studies have suggested that psychological stress can adversely influence antitumor immunity via neuroendocrine pathways, including the HPA axis and SAM system activation.^13^^,^^33^ For instance, Fraterman et al demonstrated that pretreatment ED in melanoma patients was associated with reduced responses to ICIs, suggesting that ED may impair immune activation through alterations in cytokine profiles or immune cell populations.^34^ Similarly, a recent prospective study involving patients with advanced non-small-cell lung cancer revealed a significant association between ED and unfavorable clinical outcomes, such as a shorter PFS, a lower ORR, and a reduced OS. This study also reported elevated cortisol levels in patients with ED, further linking psychological stress to immune suppression and impaired treatment efficacy.^35^ Our study extends these findings to rHGG, characterized by an immunologically “cold” microenvironment. While previous research has primarily focused on cancers with inflamed tumor microenvironments, such as melanoma, our results suggest that ED may exert an even more pronounced effect in gliomas, where baseline immune activation is inherently limited.^36^^,^^37^

The mechanisms linking ED to reduced ICB efficacy in glioma patients likely involve both systemic and localized immune modulation. ED can induce prolonged activation of the HPA axis, resulting in increased glucocorticoid secretion, known to suppress antitumor immunity by inhibiting T-cell proliferation and enhancing immune evasion.^38^^,^^39^ Sustained activation of the sympathetic nervous system may lead to increased catecholamine secretion, creating a tumor microenvironment that suppresses immune responses by stimulating regulatory T cells and myeloid-derived suppressor cells.^40–43^ Cui and Kang demonstrated that chronic stress reduces CCL3 secretion, a chemokine critical for maintaining effective antitumor immune responses. Decreased CCL3 leads to a more immunosuppressive microenvironment in gliomas, potentially hindering immune cell infiltration and antitumor activity.^44^ Additionally, key signaling pathways such as AXON guidance mediate this effect. AXON guidance genes, including SEMA4F, promote glioma cell infiltration by facilitating bidirectional signaling with neurons and remodeling peritumoral synapses.^45^ This pathway is likely exacerbated in ED patients by stress-related immune-modulating molecules, such as glucocorticoids, which heighten neuronal activity and tumor invasiveness. Recent findings reveal that the body-brain neural circuit, mediated by vagal signaling and specific brainstem neurons, plays a pivotal role in modulating peripheral inflammatory responses, maintaining immune balance in various immune dysregulation disorders, and underscoring the central nervous system’s critical function in regulating peripheral immune environments.^15^ Such a body-brain axis could represent a pathway through which ED impacts tumor immunotherapy efficacy, particularly in “cold” tumors like gliomas. The neuroimmune interactions triggered by ED through similar body-brain signaling mechanisms may lead to an immunosuppressive microenvironment, thereby influencing therapeutic outcomes in glioma patients. Our integrative findings including elevated systemic inflammatory markers, reduced TILs, high TISF-TMB, and impaired ctDNA clearance, collectively suggest a multifaceted immunosuppressive phenotype in ED patients, corresponding preclinical and clinical observations in other related studies.^46^^,^^47^

Based on neuropathological observations, we cautiously speculate that the immune responses observed in this trial may be at least partially immune-mediated. Notably, patients without ED showed an overall increase in intratumoral CD3+, CD4+, CD8+, CD68+, and CD163+ immune cells following treatment, suggesting a potentially enhanced local immune activation. In contrast, patients with ED exhibited either reduced or minimal changes in these immune populations, hinting at a possible state of immune inertia or suppression. Although limited by small sample size, this pattern echoes our systemic findings of elevated NLR and impaired ctDNA clearance in the ED group, and may reflect a multifaceted impact of psychological distress on both systemic and local immunity. Interestingly, CD163+ macrophage infiltration increased post-treatment in all patients, regardless of ED status. This uniform upregulation could signify a shared escape adaptation of recurrent gliomas, in which enrichment of CD163+ tumor-associated macrophages, frequently linked to the M2 phenotype, contributes to sustained immunosuppression despite therapeutic intervention.^48^

We acknowledge this combined treatment regimen is not an internationally established standard-of-care regimen at recurrence. In our setting, treatment selection at recurrence is shaped by local formulary and reimbursement constraints, and several agents commonly used elsewhere are not routinely available or not incorporated into local guideline-based pathways. Accordingly, our cohort reflects a pragmatic regional practice pattern, which should be considered when interpreting the external generalizability of our findings. We therefore adopted this biologically motivated approach to leverage anti-VEGF-mediated microenvironmental modulation as a potential means to support immune engagement. Mech­anistically, VEGF-driven vascular dysfunction and VEGF-associated immunosuppressive signaling may impede immune-cell trafficking.^49^ The concept of a dose-dependent vascular normalization window supports the use of appropriately dosed anti-VEGF therapy to transiently improve vascular function and immune accessibility, whereas excessive VEGF blockade may over-prune vessels and impair the delivery and efficacy of concomitant therapies.^50^^,^^51^ These microenvironmental considerations further emphasize the importance of identifying clinical factors—such as ED—that may be associated with impaired immune activation and reduced therapeutic benefit.

Given the high prevalence of ED in patients with recurrent glioma, who often experience significant psychological challenges such as anxiety and depression post-diagnosis, integrating psychosocial support into standard clinical care protocols could potentially improve treatment outcomes.^52^ Routine ED screening using validated assessment tools like the PHQ-9 and GAD-7 could facilitate early identification of at-risk patients.^29–31^ Targeted interventions, including cognitive behavioral therapy or pharmacological treatments, may mitigate the negative impact of ED on immune function.^53^ Moreover, our findings suggest that assessing ED may serve as a non-invasive biomarker, enabling the identification of patients most likely to respond positively to ICB therapy. This could facilitate more tailored treatment strategies for individual patients.

This study has several limitations. First, its observational design limits the ability to establish a definitive causal relationship between ED and ICB efficacy, despite the use of propensity score matching to reduce potential confounding. Second, ED was assessed only once at baseline, without longitudinal follow-up. This limited the ability to capture fluctuations in psychological status over time, which may be relevant to treatment response. The study did not incorporate a standardized psycho-oncology pathway, and subsequent psychological interventions or psychotropic medication use were not longitudinally captured. This limits our ability to assess how distress management may have affected ED trajectories and treatment outcomes. Third, neuroendocrine biomarkers such as cortisol, ACTH, or catecholamines were not measured. At the time of study design, there was no established consensus on optimal timing and methodology for endocrine sampling in glioma patients undergoing immunotherapy, limiting the feasibility of such measurements in this context. Finally, the single-center nature of this study may restrict the generalizability of our findings to broader populations with different demographic or healthcare characteristics.

Future studies should focus on identifying the precise molecular pathways through which ED influences the tumor immune microenvironment in gliomas. Greater attention should be directed toward the regulatory mechanisms of neuron-tumor cell interactions on the glioma immune microenvironment. Longitudinal studies incorporating serial biopsies or advanced imaging techniques could provide deeper insights into the dynamic immune changes and cytokine profiles associated with ED. Additionally, clinical trials investigating the effectiveness of psychosocial interventions alongside ICB therapy could help determine whether alleviating ED improves immunotherapy outcomes. Expanding this research to a multicenter setting would enhance the robustness of findings and support their broader applicability.

In conclusion, this study provides prospective observational evidence suggesting an association between ED and reduced immunotherapy efficacy and survival outcomes in patients with rHGG. Patients with ED experienced reduced OS and PFS, as well as lower response rates to immunotherapy. The findings emphasize the importance of incorporating psychological factors into the management of glioma patients, particularly those receiving immunotherapy. Integrating psychosocial support measures may enhance the therapeutic response to ICB and improve patient outcomes. Future research should further elucidate the mechanisms by which ED affects tumor immunity and evaluate interventions to mitigate these effects in clinical settings.

## Supplementary Material

vdag040_Supplementary_Data

## Data Availability

Data underlying this article is available upon request per Alliance procedures.
