# Supplementary material for: Emotional distress impairs immune checkpoint blockade efficacy in recurrent high-grade glioma: Insights from tumor in situ fluid analysis
Source: Neurooncol Adv. 2026 Feb 16;8(1):vdag040. doi: 10.1093/noajnl/vdag040 (PMC12952919; doi:10.1093/noajnl/vdag040)
Supplement: vdag040_Supplementary_Data [file vdag040_supplementary_data.zip › Supplementary_Figures.docx]

**

Supplementary Figure 1: Univariate and multivariate Cox regression analyses of overall survival (OS) predictors. Related to Figure 2.**

Forest plots displaying HR for various demographic, clinical, and molecular variables. (Left) Univariate Cox regression analysis. (Right) Firth-adjusted multivariate Cox regression analysis. HR: hazard ratios.

**Supplementary Figure**
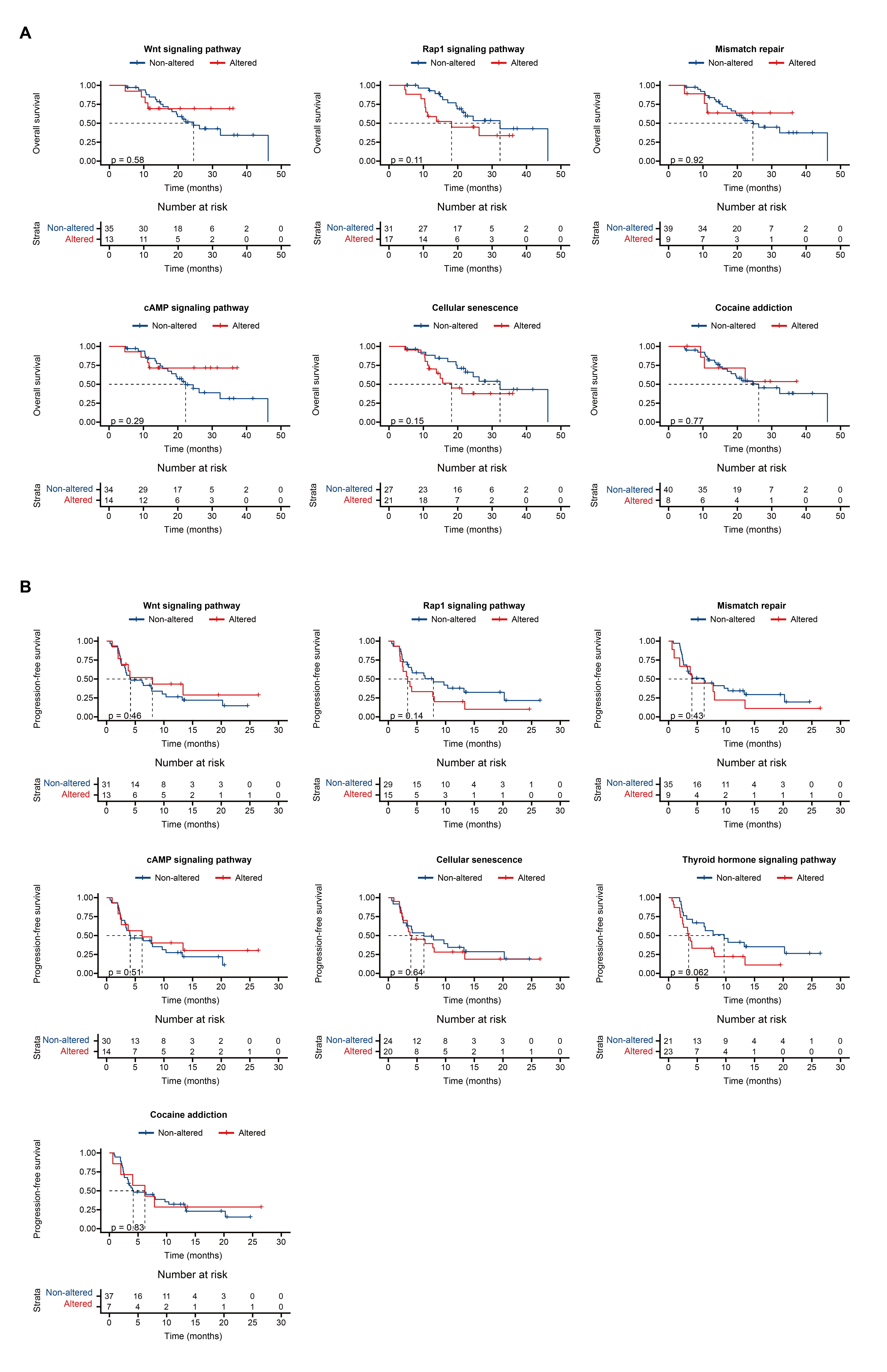
**2: Kaplan-Meier survival analysis for pathway alterations in OS and PFS. Related to Figure 4.**

**(A)** Kaplan-Meier estimate of OS for patients with and without alterations in specific pathways. **(B)** Kaplan-Meier estimate of PFS for patients with and without alterations in specific pathways. The p-value was determined by Log-rank test. OS: overall survival; PFS: progression-free survival.

**Supplementary Figure**

**3: Association of TISF-ctDNA MVAF with ED and survival outcomes**

**(A)** Comparison of TISF-ctDNA MVAF between ED and No ED groups. **(B)** Kaplan-Meier analysis of OS for patients with MVAF-H and MVAF-L levels. **(C)** Comparison of MVAF before and after treatment for ED and No ED groups. **(D)** Kaplan-Meier analysis of OS for patients with an increase or decrease in MVAF during treatment. **(E)** Kaplan-Meier analysis of OS for patients with MVAF clearance versus no MVAF clearance. The p-value was determined by Log-rank test. MVAF-H, maximal variant allele frequency-high; MVAF-L, maximal variant allele frequency-low..
