# Supplementary material for: Emotional distress impairs immune checkpoint blockade efficacy in recurrent high-grade glioma: Insights from tumor in situ fluid analysis
Source: Neurooncol Adv. 2026 Feb 16;8(1):vdag040. doi: 10.1093/noajnl/vdag040 (PMC12952919; doi:10.1093/noajnl/vdag040)
Supplement: vdag040_Supplementary_Data [file vdag040_supplementary_data.zip › Supplementary_Methods.docx]

**SUPPLEMENTARY METHODS**

*Participants eligibility criteria*

Including criteria:

(1) Age ≥18 years; (2) Karnofsky performance score (KPS) ≥ 70; (3) A histologically confirmed diagnosis of high-grade gliomas with disease progression, as evidenced by imaging; (4) Receiving a standardized first-line treatment regimen post-surgery; (5) No prior history of mental illness, substance abuse, or alcohol misuse; (6) Ability to independently communicate, read, and write; (7) Willingness and capacity to adhere to the study protocol, as confirmed by the investigator’s signed informed consent.

Excluding criteria:

(1) More than two episodes of glioma recurrence; (2) The presence of extracranial metastasis, extensive leptomeningeal involvement, or tumors predominantly located in the brainstem or spinal cord; (3) Evidence of conditions interfering with stress axis measurement (e.g., chronic corticosteroid use ≥ 3 months before study entry for non-glioma diseases; dexamethasone use in glioma context is permitted); (4) Severe mental illness prior to glioma diagnosis; (5) Participation in studies involving investigational drugs; (6) Pregnancy or breastfeeding; (7) Inability to follow study procedures due to language barriers, psychological disorders, neurological deficits interfering with walking tests, dementia, or confusional states.

*Assessment of quality of life (QoL)*

Quality of life (QoL) was evaluated using the EORTC QLQ-C30, a widely recognized instrument for assessing health-related QoL in oncology.^1^ This tool consists of 30 questions that span 15 distinct domains, including five functional areas, three symptom-related factors, one domain for global health status/QoL, and six additional single-item domains. The scoring system utilizes a standardized range from 0 to 100. Higher values in the global health status/QoL and functional categories signify improved quality of life, while elevated scores in the symptom categories correspond to greater symptom severity. QoL assessments were conducted simultaneously with evaluations of ED.

*DNA extraction and library preparation*

Genomic DNA was isolated from leukocytes using the MagMAX™ DNA Multi-Sample Ultra Kit (Thermo). Circulating free DNA (cfDNA) from TISF was extracted with the MagMAX™ Cell-Free DNA Isolation Kit (Thermo) in accordance with the manufacturer’s guidelines. DNA concentrations were determined using the Qubit dsDNA HS Assay Kit (Thermo) on a Qubit fluorometer, and its quality was analyzed with the Agilent 4200 TapeStation (Agilent). For preparing the libraries, both commercial and custom-designed probes were used in the hybridization capture process. DNA samples (15–200 ng) were fragmented to sizes between 200 and 350 bp. Paired-end adapters specific to the Illumina platform were custom-created by SimcereDx. For fragmented DNA, end-repair, A-tailing, and adapter ligation were carried out using the KAPA HyperPlus DNA Library Preparation Kit (Roche Diagnostics). In contrast, the VAHTS™ Universal DNA Library Preparation Kit for Illumina® (Vazyme) was employed for cfDNA processing. Unligated adapters were removed through size selection performed with Agencourt AMPure XP beads (Beckman Coulter). The ligated DNA products were then amplified by PCR to create pre-libraries for subsequent hybridization. Finally, the libraries were quantified once more using the Qubit fluorometer and their quality was verified with the Agilent 4200 TapeStation.

*Library sequencing and bioinformatics analysis*

DNA libraries were sequenced using the Illumina NovaSeq6000 platform, generating paired-end reads of 150 bp.^2^ Base calling outputs were converted into FASTQ files. Adapter sequences and low-quality bases were trimmed using fastp (v.2.20.0) (PMID: 30423086). Alignment of the sequences was carried out with the BWA-MEM algorithm (v.0.7.17) against the hg19 GRCh37 reference genome (UCSC) (PMID: 30824715). Deduplication with error correction was employed to remove duplicate PCR reads. Single nucleotide variants (SNVs) and indels were identified and annotated using VarDict (v.1.5.7) (PMID: 27060149) and InterVar (PMID: 28132688). Variant filtering was conducted by comparing against common SNPs in public datasets, such as the 1000 Genomes Project (August 2015) and the ExAC Browser (v.0.3). Copy number variations (CNVs) and gene fusions were analyzed with CNVkit (v.1.1) (PMID: 27100738) and Factera (v.1.4.4) (PMID: 25143292), respectively. Tumor mutational burden (TMB) was computed by counting somatic coding mutations, including both base substitutions and indels, per megabase of the analyzed genome. For the 551-gene cancer panel, TMB was estimated by summing all base substitutions and indels within coding regions of target genes, excluding synonymous mutations, variants with an allele frequency (AF) under 0.02, and mutations recorded as known somatic alterations in the COSMIC database.

*Immunohistochemistry and quantitative analysis*

Formalin-fixed paraffin-embedded (FFPE) tumor tissues from paired pre- and post-treatment resections of rHGG (n=4 patients) were sectioned at 4 µm thickness. Immunostaining was performed on a Bond IHC stainer (Leica Biosystems) using standardized protocols. The following ready-to-use antibodies were applied: CD3 (clone LN10; Leica PA0553), CD4 (clone 4B12; Leica PA0427), CD8 (clone 4B11; Leica PA0183), CD68 (clone KP1; Dako GA613), and CD163 (clone 10D6; Leica PA0443). Antigen retrieval used EDTA-based solution (pH 9.0) at 100°C for 20 minutes. Negative controls omitted primary antibodies; human tonsil sections served as positive controls. Quantitative assessment was performed by a board-certified neuropathologist blinded to clinical data. Six randomly selected 0.25 mm² fields per slide at the tumor invasion front were captured at 400× magnification (Olympus BX53). Immune cell quantification was conducted using ImageJ software (v1.53k, NIH).

**SUPPLEMENTARY METHODS REFERENCES**

**1.** Martinelli F, Quinten C, Maringwa JT, et al. Examining the relationships among health-related quality-of-life indicators in cancer patients participating in clinical trials: a pooled study of baseline EORTC QLQ-C30 data. *Expert Rev. Pharmacoecon. Outcomes Res.* 2011; 11(5):587-599.

**2.** Modi A, Vai S, Caramelli D, Lari M. The Illumina Sequencing Protocol and the NovaSeq 6000 System. In: Mengoni A, Bacci G, Fondi M, eds. *Bacterial Pangenomics, 2 Edition:Methods and Protocols.* Vol 2242: Humana Press Inc, 999 Riverview Dr, Ste 208, Totowa, Nj 07512-1165 USA; 2021:15-42.
