# Supplementary material for: Emotional distress impairs immune checkpoint blockade efficacy in recurrent high-grade glioma: Insights from tumor in situ fluid analysis
Source: Neurooncol Adv. 2026 Feb 16;8(1):vdag040. doi: 10.1093/noajnl/vdag040 (PMC12952919; doi:10.1093/noajnl/vdag040)
Supplement: vdag040_Supplementary_Data [file vdag040_supplementary_data.zip › Supplementary_Tables.docx]

| **Supplementary Table 1 \| The binary logistic regression analysis for evaluating the likelihood of emotional distress** | | | | |
| --- | --- | --- | --- | --- |
| **Characteristics** |  | ***P* value** ^a^ | **OR** | **95% CI** |
| **Age (years)** | <55 |  | 1(Reference) |  |
|  | ≥55 | 0.399 | 0.508 | 0.106-2.448 |
| **Sex** | Male |  | 1(Reference) |  |
|  | Female | 0.981 | 1.018 | 0.228-4.554 |
| **KPS** | 70-80 |  | 1(Reference) |  |
|  | 90-100 | 0.038 | 0.219 | 0.052-0.920 |
| **IDH status** | Wild |  | 1(Reference) |  |
|  | Mutant | 0.830 | 0.840 | 0.171-4.118 |
| **MGMT promoter methylation** | No |  | 1(Reference) |  |
|  | Yes | 0.921 | 1.078 | 0.247-4.701 |
|  | Not-detected | 0.324 | 0.391 | 0.060-2.534 |
| **Radiation** | No |  | 1(Reference) |  |
|  | Yes | 0.097 | 0.320 | 0.083-1.232 |
| **Tumor size at baseline** |  | 0.335 | 1.017 | 0.983-1.053 |
| **Baseline steroid use** | No |  | 1(Reference) |  |
|  | Yes | 0.833 | 1.181 | 0.252-5.531 |
| **Extent of surgery** | Complete resection |  | 1(Reference) |  |
|  | Partial resection | 0.380 | 1.895 | 0.455-7.894 |
| **Smoking** | No |  | 1(Reference) |  |
|  | Yes | 0.103 | 4.293 | 0.744-24.772 |
| **Hypertension/**  **diabetes** | No |  | 1(Reference) |  |
|  | Yes | 0.557 | 0.665 | 0.170-2.600 |
| **Educational level** | <High school |  | 1(Reference) |  |
|  | ≥High school | 0.695 | 0.761 | 0.195-2.973 |
| **Marital status** | Married |  | 1(Reference) |  |
|  | Others | 0.163 | 10.666 | 0.385-295.493 |
| **Job status** | Unemployed |  | 1(Reference) |  |
|  | Employed | 0.697 | 1.387 | 0.267-7.208 |
| **Caregiver** | Spouse |  | 1(Reference) |  |
|  | Others | 0.530 | 0.571 | 0.099-3.281 |
| **Residence** | Country |  | 1(Reference) |  |
|  | City | 0.194 | 0.356 | 0.075-1.692 |
| **Monthly income**  **(CNY)** | <5,000 |  | 1(Reference) |  |
|  | ≥5,000 | 0.235 | 2.755 | 0.517-14.670 |
| **BMI (kg/m^2^)** | <24 |  | 1(Reference) |  |
|  | ≥24 | 0.261 | 2.035 | 0.590-7.023 |
| ^a^P values were calculated using a two-sided binary logistic regression. Dependent variable = emotional distress | | | | |

| **Supplementary Table 2 \| Baseline characteristics after propensity-score matching** | | | | | |
| --- | --- | --- | --- | --- | --- |
| **Characteristics** |  | **No ED**  **(n = 29)** | **ED**  **(n = 29)** | **Total (n = 58)** | **P value** |
| **Age (years), *n* (%)** | <55 | 13(44.8) | 17(58.6) | 30(51.7) | 0.293 |
|  | ≥55 | 16(55.2) | 12(41.4) | 28(48.3) |  |
| **Sex, *n* (%)** | Male | 17(58.6) | 19(65.5) | 36(62.1) | 0.588 |
|  | Female | 12(41.4) | 10(34.5) | 22(37.9) |  |
| **KPS, *n* (%)** | 70-80 | 10(34.5) | 10(34.5) | 20(34.5) | 1.000 |
|  | 90-100 | 19(65.5) | 19(65.5) | 38(65.5) |  |
| **Histopathologic diagnosis, *n* (%)** | Glioblastoma, IDH-wildtype | 24(82.8) | 26(89.7) | 50(86.2) | 0.706 ^a^ |
|  | Astrocytoma, IDH-mutant | 4(13.8) | 3(10.3) | 7(12.1) |  |
|  | Oligodendroglioma, IDH-mutant | 1(3.4) | 0(0.0) | 1(1.7) |  |
| **Primary tumor location, *n* (%)** | Frontal lobe | 11(37.9) | 11(37.9) | 22(37.9) | 0.743 ^a^ |
|  | Temporal lobe | 8(27.6) | 11(37.9) | 19(32.8) |  |
|  | Parietal lobe | 5(17.2) | 5(17.2) | 10(17.2) |  |
|  | Thalamus | 3(10.3) | 1(3.4) | 4(6.9) |  |
|  | Occipital lobe | 1(3.4) | 0(0.0) | 1(1.7) |  |
|  | Insular lobe | 1(3.4) | 0(0.0) | 1(1.7) |  |
|  | Brainstem | 0(0.0) | 1(3.4) | 1(1.7) |  |
| **MGMT promoter methylation, *n* (%)** | No | 8(27.6) | 13(44.8) | 21(36.2) | 0.371 |
|  | Yes | 8(27.6) | 7(24.1) | 15(25.9) |  |
|  | Not-detected | 13(44.8) | 9(31.0) | 22(37.9) |  |
| **Radiation, *n* (%)** | No | 18(62.1) | 21(72.4) | 39(67.2) | 0.401 |
|  | Yes | 11(37.9) | 8(27.6) | 19(32.8) |  |
| **Tumor size at baseline (mm)** | Median | 30.6 | 25.4 | 29.5 | 0.774 ^b^ |
|  | Range | 15.1-77.6 | 8.9-81.9 | 8.9-81.9 |  |
| **Baseline steroid use, *n* (%)** | No | 22(75.9) | 21(72.4) | 43(74.1) | 0.764 |
|  | Yes | 7(24.1) | 8(27.6) | 15(25.9) |  |
| **Extent of surgery, *n* (%)** | Complete resection | 21(72.4) | 16(55.2) | 37(63.8) | 0.274 ^a^ |
|  | Partial resection | 8(27.6) | 12(41.4) | 20(34.5) |  |
|  | Unable to assess | 0(0.0) | 1(3.4) | 1(1.7) |  |
| **Smoking, *n* (%)** | No | 23(79.3) | 21(72.4) | 44(75.9) | 0.539 |
|  | Yes | 6(20.7) | 8(27.6) | 14(24.1) |  |
| **Hypertension/**  **diabetes, *n* (%)** | No | 19(65.5) | 20(69.0) | 39(67.2) | 0.780 |
|  | Yes | 10(34.5) | 9(31.0) | 19(32.8) |  |
| **Educational level,**  ***n* (%)** | <High school | 13(44.8) | 13(44.8) | 26(44.8) | 1.000 |
|  | ≥High school | 16(55.2) | 16(55.2) | 32(55.2) |  |
| **Marital status, *n* (%)** | Married | 28(96.6) | 24(82.8) | 52(89.7) | 0.194 ^a^ |
|  | Others | 1(3.4) | 5(17.2) | 6(10.3) |  |
| **Job status, *n* (%)** | Unemployed | 25(86.2) | 23(79.3) | 48(82.8) | 0.487 |
|  | Employed | 4(13.8) | 6(20.7) | 10(17.2) |  |
| **Caregiver, *n* (%)** | Spouse | 11(37.9) | 14(48.3) | 25(43.1) | 0.426 |
|  | Others | 18(62.1) | 15(51.7) | 33(56.9) |  |
| **Residence, *n* (%)** | Country | 21(72.4) | 22(75.9) | 43(74.1) | 0.764 |
|  | City | 8(27.6) | 7(24.1) | 15(25.9) |  |
| **Monthly income**  **(CNY), *n* (%)** | <5,000 | 24(82.8) | 21(72.4) | 45(77.6) | 0.345 |
|  | ≥5,000 | 5(17.2) | 8(27.6) | 13(22.4) |  |
| **BMI (kg/m2), *n* (%)** | <24 | 19(65.5) | 13(44.8) | 32(55.2) | 0.113 |
|  | ≥24 | 10(34.5) | 16(55.2) | 26(44.8) |  |
| ^a^The histopathologic diagnosis, primary tumor location, extent of surgery, and marital status were analyzed using two-sided Fisher’s exact tests, and the proportions of all other categorical characteristics were analyzed using two-sided χ2 tests. ^b^Tumor size at baseline was compared between groups using the Mann-Whitney U test. | | | | | |

| Supplementary Table 3 \| The association between emotional distress and quality of life | | | |
| --- | --- | --- | --- |
| **Domains** | **No ED**  **(*n* = 39)** | **ED**  **(*n* = 36)** | ***P* value ^a^** |
| Global health | 83.3 (66.7-83.3) | 50.0 (35.4-58.3) | <0.001 |
| **Functions** |  |  |  |
| Physical functioning | 86.7 (80.0-93.3) | 63.3 (46.7-80.0) | <0.001 |
| Role functioning | 100.0 (66.7-100.0) | 66.7 (50.0-95.8) | <0.001 |
| Emotional functioning | 91.7 (83.3-100.0) | 75.0 (58.3-83.3) | <0.001 |
| Cognitive functioning | 66.7 (66.7-83.3) | 50.0 (33.3-66.7) | <0.001 |
| Social functioning | 83.3 (66.7-100.0) | 50.0 (33.3-66.7) | <0.001 |
| **Symptoms** |  |  |  |
| Fatigue | 33.3 (22.2-33.3) | 33.3 (33.3-66.7) | 0.001 |
| Nausea and vomiting | 0.0 (0.0-0.0) | 0.0 (0.0-29.2) | 0.043 |
| Pain | 0.0 (0.0-16.7) | 16.7 (0.0-33.3) | 0.010 |
| Dyspnea | 0.0 (0.0-0.0) | 0.0 (0.0-33.3) | 0.141 |
| Insomnia | 0.0 (0.0-33.3) | 33.3 (0.0-33.3) | 0.008 |
| Appetite loss | 0.0 (0.0-33.3) | 33.3 (0.0-33.3) | 0.002 |
| Constipation | 0.0 (0.0-33.3) | 0.0 (0.0-33.3) | 0.580 |
| Diarrhea | 0.0 (0.0-0.0) | 0.0 (0.0-0.0) | 0.881 |
| Financial difficulties | 33.3 (33.3-66.7) | 66.7 (33.3-100.0) | <0.001 |
| ^a^P values were calculated using a two-sided Mann-Whitney U test. The data were described by the median (IQR). | | | |
